# Specific antigen-based stratification of membranous nephropathy in patients after haematopoietic stem cell allotransplantation - a case series and literature review

**DOI:** 10.1186/s12882-024-03675-y

**Published:** 2024-08-08

**Authors:** Ines Bosnić Kovačić, Matija Matošević, Mario Laganović, Živka Dika, Margareta Fištrek Prlić, Ema Ivandić, Marijana Ćorić, Stela Bulimbašić, Nadira Duraković, Zinaida Perić, Lana Desnica, Radovan Vrhovac, Bojan Jelaković, Sanjeev Sethi, Ivana Vuković Brinar

**Affiliations:** 1 Department of Nephrology and Dialysis, Clinical Hospital Sveti Duh, Zagreb, Croatia; 2 Institute of Emergency Medicine of Zagreb County, Velika Gorica, Croatia; 3 Department of Nephrology, University Hospital Merkur, Zagreb, Croatia; 4 School of Medicine, University of Zagreb, Zagreb, Croatia; 5 Department of Nephrology, Arterial Hypertension, Dialysis and Transplantation, University Hospital Centre Zagreb, Zagreb, Croatia; 6 Department of Pathology and Cytology, University Hospital Centre Zagreb, Zagreb, Croatia; 7 Department of Haematology, University Hospital Centre Zagreb, Kišpatićeva 12, Zagreb, Croatia; 8 Department of Laboratory Medicine and Pathology, Mayo Clinic, Rochester, MN USA

**Keywords:** FAT1, Hematopoietic stem cell transplant, Membranous nephropathy, PCSK6

## Abstract

**Background:**

Nephrotic syndrome (NS) is a rare complication that can occur after haematopoietic stem cell transplantation (HSCT). In patients with membranous nephropathy (MN) who have undergone allogeneic HSCT, a new antigen called protocadherin FAT1 has been identified. Our objective is to present a case series of MN patients after HSCT with a novel antigen-based stratification.

**Case presentations:**

Patients who developed full-blown NS due to MN after an HSCT were enrolled in the University Hospital Centre Zagreb study. The first two patients were treated with an HSCT for acute myeloid leukaemia, and both developed NS after cessation of graft versus host disease (GVHD) prophylaxis. The first patient had reduced kidney function, while the second had completely preserved function. Kidney biopsy showed MN with only subepithelial deposits. A thorough examination revealed that there was no secondary cause of the disease. The patients achieved complete remission after undergoing immunosuppression treatment. The third patient underwent HSCT for acute lymphoblastic leukaemia. He developed both acute and chronic GVHD and also experienced avascular hip necrosis. After sixteen years, the patient developed NS with preserved kidney function. The kidney specimen showed membranous nephropathy (MN) with mesangial and subepithelial deposits. Extensive research was conducted, but no secondary cause for the MN was detected. All three cases tested negative for anti-PLA2R antibodies. Biopsy tissue samples were analysed using laser microdissection and tandem mass spectrometry of glomeruli for the detection of different specific antigens. Patients one and two tested positive for FAT1, whereas patient three tested positive for PCSK6.

**Conclusions:**

MN can develop at various time intervals after HSCT. Specific antigen testing can help establish the relationship between MN and HSCT. In the future, serum testing for anti-FAT1 antibodies in HSCT patients could be significant in diagnosing FAT1-associated MN, similar to how anti-PLA2R antibodies are significant in diagnosing PLA2R-associated MN.

**Supplementary Information:**

The online version contains supplementary material available at 10.1186/s12882-024-03675-y.

## Background

Medical science has made significant progress in treating patients who require hematopoietic stem cell transplantation (HSCT). However, even with better supportive care, human leukocyte antigen (HLA) matching, and control of pre- and post-transplant complications, chronic graft versus host disease (cGVHD) remains a potentially life-threatening immunological complication that can develop in up to 80% of transplant recipients. The condition is treated with immunosuppressive therapy (IS) [1, 2]. One of the rare complications of HSCT is the development of renal disease, especially nephrotic syndrome (NS) due to glomerulonephritis [3–6]. A systematic review of post-HSCT patients who presented with NS revealed that 116 cases were published in the English literature between 1988 and 2015. Membranous nephropathy (MN) was the most frequent pathology (65.5%), followed by minimal change disease (19%) [6].

MN is one of the most common causes of adult-onset NS and also the most common glomerular disease in conjunction with HSCT [7]. The primary distinction in MN is based on its aetiology, which can be either primary or secondary. This distinction is crucial in determining the appropriate treatment approach.

Taking that into consideration, it is of utmost importance to distinguish whether MN after HSCT is a condition that merits treatment with IS or not. There are two explanations for the connection between HSCT and MN. Brukamp et al. suggest that MN may be a renal manifestation of GVHD [5] while Abudayyeh et al. and Mrabet et al. suggest that MN may be a secondary result of an autoimmune response to an allogeneic transplant [3, 7]. Taking that into consideration, IS is a viable treatment option for MN after HSCT [2, 5, 6, 8].

Recent advances in the field of specific antigens associated with different forms of MN enable an individualized approach for patients with MN [9–11]. The protocadherin FAT atypical cadherin 1 (FAT1) antigen is a novel antigen identified only in patients with MN who have undergone allogeneic HSCT (aHSCT). It can specifically distinguish HSCT-related MN from other forms and helps in deciding upon the specific treatment [9–11]. As the body of literature considering renal complications involving HSCT and GVHD continues to grow, further understanding of intricate connections between these conditions is expected. As a step in that direction, we describe three cases involving HSCT and subsequent development of NS due to MN with long-term follow-up and MN-specific antigen testing. In addition to our reports, we conducted a literature review of MN cases associated with HSCT.

## Methods

After undergoing aHSCT in Croatia, patients are regularly monitored at the University Hospital Centre (UHC) Zagreb, which enables us to collect data on all Croatian patients who have undergone HSCT. Over the past 30 years, 1302 HSCTs were performed, out of which only 3 patients (0.2%) developed NS.

We present a report on three patients who developed NS after undergoing an aHSCT and were diagnosed with MN. Biopsy samples were analysed for novel target MN antigens. The patients were tested for serum anti-phospholipase A2 receptor (anti-PLA2R) antibody. A kidney biopsy was performed, and the samples were examined using light microscopy (LM), immunofluorescence (IF), and electron microscopy (EM). Routine IF staining included IgA, IgM, IgG, C1q, C3, kappa, lambda light chains, and fibrin.

Samples of biopsy tissue were sent to the Renal Pathology Laboratory at the Department of Laboratory Medicine and Pathology at Mayo Clinic to identify FAT1 antigen. The biopsy samples were dissected from paraffin-embedded tissue to extract glomeruli. The glomeruli were then digested by trypsin and analysed using mass spectrometry to identify and semi-quantify the glomerular proteins, as described by Sethi et al. [10].

We have conducted a literature search to identify cases of membranous nephropathy/nephritis in patients who have undergone HSCT. The search was performed on Scopus and MEDLINE/PubMed databases in the English language, up to July 2023, using the keywords “membranous nephropathy” and “hematopoietic stem cell transplant” as per the published guidelines for narrative reviews.

### Case presentations

**Fig. 1:**
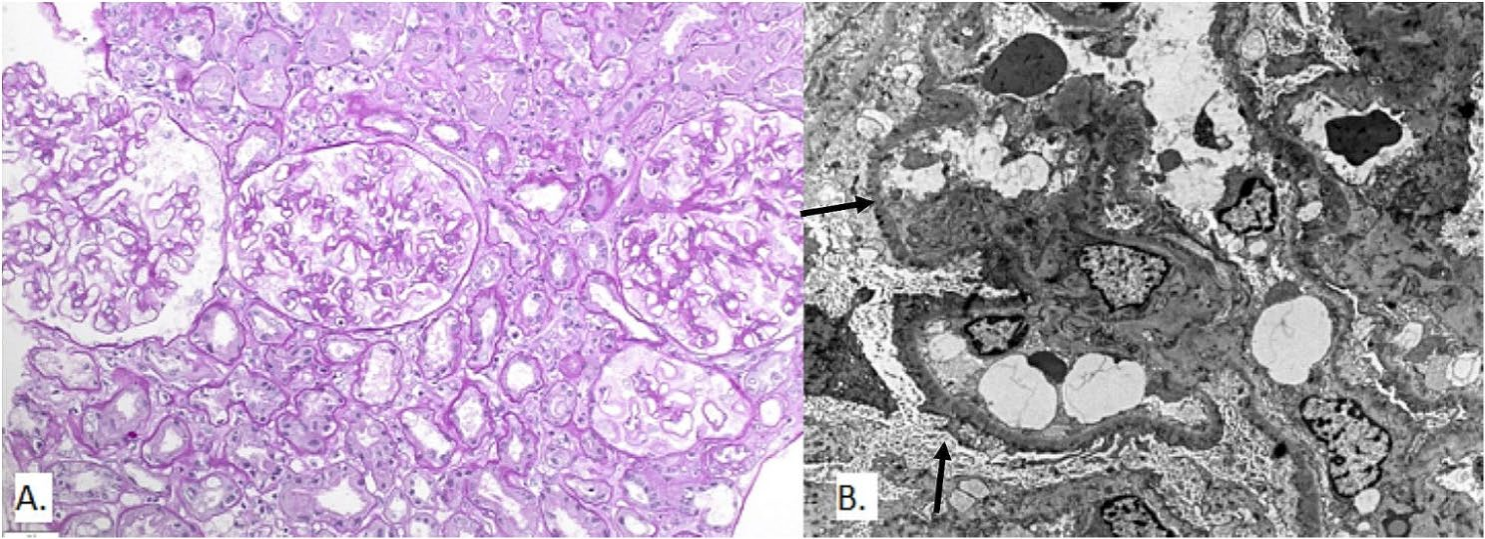
Kidney biopsy findings in Patient 1. **A** Light microscopy: Glomeruli with discreet thickening of glomerular capillary wall. (PAS stain, magnification 200x). **B** Electron microscopy shows many subepithelial deposits (arrows) and extensive effacement of the podocyte foot processes with prominent microvillous hypertrophy (original magnification ×1800)

The following case report describes a 39-year-old Caucasian (Patient 1.) woman who underwent an aHSCT in 2009. The transplant was necessary due to her acute myelogenous leukaemia (AML) M5, for which she received bone marrow stem cells from a related donor. Before the transplant, she was given a conditioning regimen consisting of cyclophosphamide (CYC), busulfan, cisplatin, and methotrexate (MTX). She also received GVHD prophylaxis for six months and did not experience any acute GVHD (aGVHD). However, after discontinuing immunosuppression therapy, she developed liver GVHD, which was treated with mycophenolate mofetil (MMF). Two years after the HSCT, the patient developed NS with proteinuria of 13 g/day, serum protein of 47 g/L, total cholesterol of 18 mmol/L, and decreased renal function (creatinine 103 µmol/L, eGFR CKD EPI 58 mL/min/1.73m^2^) (Table 1.). A kidney biopsy was performed (Fig. 1), which revealed a mildly to moderately thickened glomerular basement membrane (GBM). Immunoglobulin G (IgG) deposits with a granular pattern and intensity of 3 + were observed along the glomerular basement membrane (GBM), while C3 deposits were 1+ (Table 2). EM showed subepithelial electron-dense deposits, GBM spikes, and intramembranous deposits. No mesangial deposits were found, which was suggestive of primary MN, but serum anti-PLA2R was negative. The workup for secondary causes included several tests such as multi-slice computed tomography (CT) of the neck, thorax, abdomen, and pelvis, colonoscopy, esophagogastroduodenoscopy, echocardiography, immunological test for antinuclear antibody (ANA), as well as serology for hepatitis B, C, and human immunodeficiency virus (HIV).

**Table 1:** Clinical characteristics and outcomes of three patients with membranous nephropathy after haematopoietic stem cell transplantation

Patient Number	Age at NS onset	Sex	Transplant	Primary Disease	Years to MN	Initial Serum Creatinine (µmol/L)	Initial Proteinuria (g/day)	Outcome (months after NS onset)	Follow-up Serum Creatinine (µmol/L)	Follow-up Proteinuria (g/day)
1	41	F	BMSCT	AML	2.2	103	11.8	147	57	0.2
2	56	M	PBSCT	AML	0.75	81	4	78	84	0.1
3	45	M	PBSCT	ALL	26	72	12	24	67	1

ALL – acute lymphoblastic leukaemia, AML- acute myeloid leukaemia, BMSCT - Bone Marrow Stem Cell Transplant, F- female, M- male, MN- membranous nephropathy, NS- nephrotic syndrome, PBSCT - Peripheral Blood Stem Cell Transplant

After excluding secondary causes, renal GVHD was suspected and treated with a tapering dose of oral prednisone at 0.8 mg/kg and oral cyclophosphamide (CYC) at 1 mg/kg/day in two divided doses (bid). Additionally, conservative antiproteinuric therapy was administered with an angiotensin-converting enzyme (ACE) inhibitor. Immunosuppressive therapy was discontinued after six months due to infection complications, specifically maxillary sinus empyema, which required multiple operative treatments including ethmoidectomy, nasal duct decompression, and bilateral antrostomy formation. At that point, partial remission was achieved with proteinuria of 5.3 g/day, followed by complete remission in January 2013. This remission has been sustained throughout the entire follow-up period of 13 years, as noted in Table 1.

**Table 2:** Renal biopsy findings of three patients with membranous nephropathy after haematopoietic stem cell transplantation

	Patient Number		
	1	2	3
**LM**			
No. of glomeruli	43	14	50
No. of sclerosed glomeruli	5	1	3
Spike formation on GBM	(+)	(−)	(+)
Endocapillary proliferative lesion	(−)	(−)	(−)
Other findings		Interstitial mononuclear infiltration foci and slight tubular damage	Nodular arteriolar hyalinosis
IFTA (%)	25%	0%	5%
**IF**			
IgG	GBM(3+)Mes(−)	GBM(3+)Mes(−)	GBM(3+)Mes(3+)
IgA	(−)	(2+)	(-)
IgM	(2+)	(2+)	(-)
C3	(1+)	(3+)	(1+)
C1q	(2+)	(−)	(-)
Kappa light chain	(2+)	(2+)	(3+)
Lambda light chain	(2+)	(2+)	(3+)
Fibrin	(1+)	(2+)	(-)
**EM**			
EDD	Subepi(+)	Subepi(+)	Subepi(+) Subendo (+) Mes(+)
TBM deposits	(-)	(-)	(-)
EM Stage (Ehrenreich Churg classification)	II-III	I	II
Serum anti-PLA2R	Negative	Negative	Negative
Antigen detected with LMD/MS	FAT1	FAT1	PCSK6

anti-PLA2R - - phospholipase A2 receptor, EDD- electron-dense deposits, EM- electron microscopy, GBM- along the glomerular basement membrane. IF- immunofluorescence, IgG, IgA, IgM- immunoglobulin G, A, and M, LM- light microscopy, LMD/MS- laser microdissection/mass spectrometry, Mes- in the mesangial space, Subendo- in the subepithelial spaces, Subepi- in the subepithelial spaces

The second case involves a 55-year-old Caucasian male (Patient 2.) who had undergone an allogeneic peripheral blood stem cell transplantation (PBSCT) from a 10/10 HLA-matched unrelated female donor due to AML M1 back in 2015. A reduced-intensity conditioning regimen was applied, including fludarabine, low-dose oral busulfan, and antithymocyte globulin (ATG).

After undergoing HSCT, AML went into complete remission. To prevent GVHD, the patient was given ATG twice, followed by cyclosporin A (CsA) and MMF for nine months. Ten months post-HSCT and one month after discontinuing CsA and MMF, the patient developed full-blown NS with oedema of lower extremities, proteinuria of 4 g/day, hypoalbuminemia (18 g/L), and preserved kidney function (creatinine 81 µmol/L, eGFR CKD EPI 93 ml/min/1.73 m^2^).

**Fig. 2:**
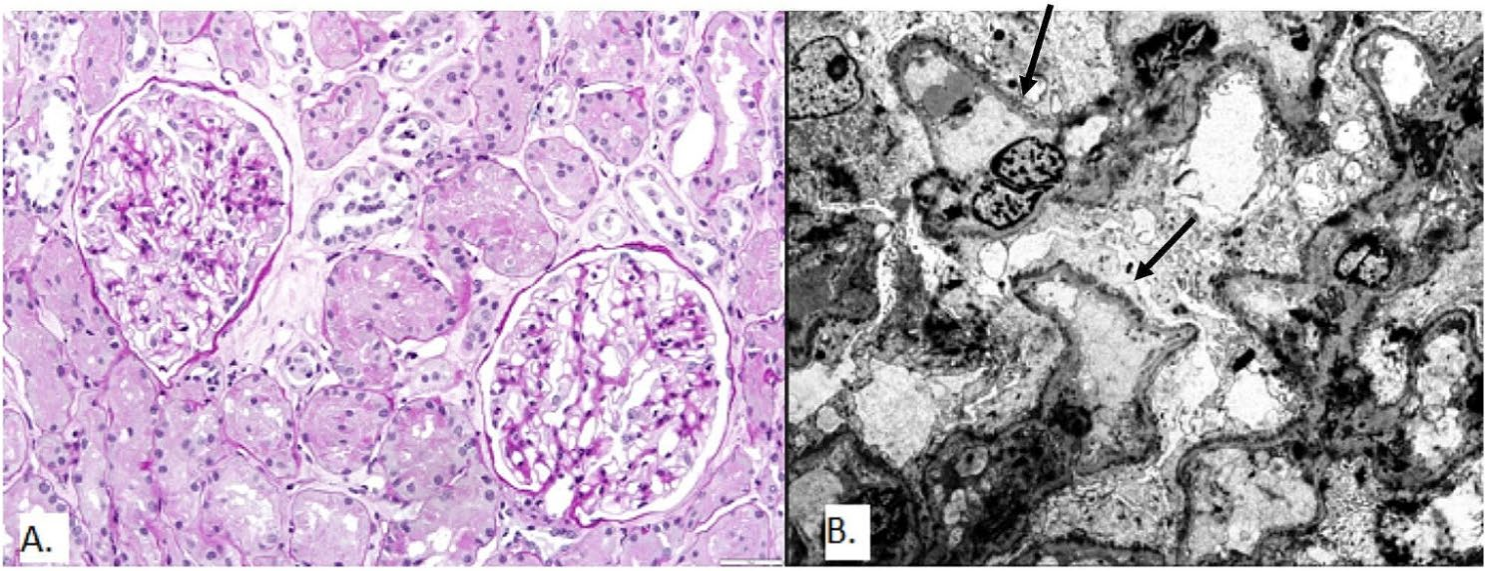
Kidney biopsy findings in Patient 2. **A** Light microscopy: Glomeruli with no GBM abnormalities (PAS stain, magnification 200x). **B** Electron microscopy reveals numerous subepithelial deposits (arrows) with diffuse effacement of the podocyte foot processes (original magnification ×1800)

The patient’s clinical characteristics and outcomes are summarized in Table 1. A kidney biopsy was conducted, as shown in Fig. 2. The biopsy revealed MN without changes in LM (Table 2). However, there were granular IgG deposits of intensity 3 + and C3 3 + along the GBM. Electron-dense deposits were found exclusively in the subepithelial region of GBM without deposits in the mesangial area.

Once again, serum anti-PLA2R was negative, and other secondary causes were excluded as described above. A haematological workup revealed a full chimera with remission of AML, so renal GVHD was suspected and as such treated with IS. He received oral methylprednisolone (MP) 1 mg/kg/day in two divided doses in a tapering manner and parenteral CYC 500 mg once per month along with conservative antiproteinuric renin-angiotensin-aldosterone system (RAAS) blockade. After treatment with two CYC applications, a urosepsis and shingles occurred, and CYC was switched to CsA 200 mg/day in two divided doses (bid). Three months after IS initiation, complete remission was achieved and CsA was tapered. During the 6.5-year follow-up period, the patient is in a sustained remission.

The patient in the third case is a Caucasian male (Patient 3.) who was born in 1974. He received aHSCT from his HLA identical brother back in 1993. The HSCT was conducted after myeloablative conditioning, which involved the use of CYC and total body irradiation, to treat his acute lymphoblastic leukaemia (ALL). Despite receiving GVHD prophylaxis with MTX, he developed aGVHD, which affected his skin, liver, and bowel. He was treated with MP, ATG, and MTX. In 1994, the patient developed aseptic necrosis of the femoral head and avascular necrosis. In the following year, 1995, he was diagnosed with chronic scleroderma-like skin and mild liver cGVHD. He was treated with MP, CsA, and radiotherapy of the thoracolumbar region, followed by azathioprine. Finally, in 2009, sixteen years after HSCT, he developed signs of oral and ocular cGVHD.

**Fig. 3:**
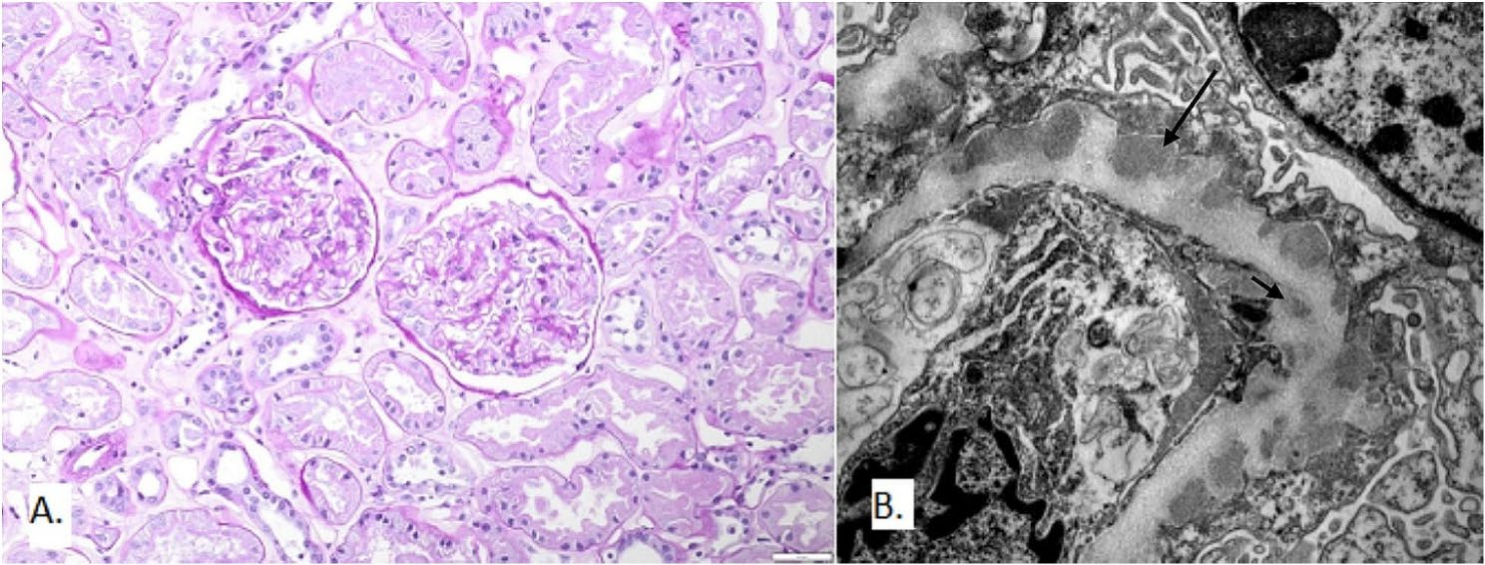
Kidney biopsy findings in Patient 3. **A** Light microscopy: Glomeruli with discreet thickening of glomerular capillary wall. (PAS stain, magnification 200x). **B** Electron microscopy: Numerous subepithelial (long arrow) and few subendothelial (short arrow) immune deposits. Podocytes show dffuse effacement of foot processes and microvilous transformation. Original magnification 12000x

In 2019, a patient who had undergone HSCT 26 years prior and was now 45 years old presented with NS and proteinuria of 12 g/day, but preserved kidney function (creatinine 72 µmol/L, eGFR CKD EPI 107 ml/min/1.73m^2^). The patient also showed worsening skin cGVHD changes. A kidney biopsy revealed thickening of GBM on light microscopy, along with diffuse granular IgG deposits of intensity 3 + and C3 1 + at GBM consistent with MN (Table 2). Electron-dense deposits were found in the subepithelial, subendothelial, and mesangial departments of glomeruli, and nodular arteriolar hyalinosis was also present (Fig. 3). Due to the pathology finding in line with secondary anti-PLA2R negative MN, the long interval from HSCT to NS onset, and a large amount of chemotherapy and immunosuppression previously applied, secondary malignant disease was suspected. However, a thorough workup did not detect a secondary cause of MN. The patient’s clinical characteristics and outcomes are summarized in Table 1. Haematological GVHD assessment showed a worsening of chronic GVHD of the skin and eyes. Despite 10 months of conservative treatment, the persistence of nephrotic proteinuria and chronic GVHD with acute GVHD of the skin led to a second kidney biopsy. Other signs of secondary MN unrelated to HSCT were present, but the biopsy showed only subepithelial electron-dense deposits. This prompted the decision to apply specific IS. The patient was administered one dose of rituximab (1000 mg intravenously), but the second dose had to be postponed due to the COVID-19 pandemic. However, partial remission was achieved in 2021 with proteinuria of 1 g/day, followed by complete remission in 2022, which has been sustained till the present.

Subsequently, biopsy samples from all patients were sent to the Mayo Clinic, to identify glomerular proteins using laser capture microdissection and tandem mass spectrometry (LCM/MS). Comprehensive results are displayed in Table 3. The first two cases were found to be positive for FAT1 antigen, which is consistent with MN related to haematopoietic stem cell transplantation [10]. The third patient was positive for PCSK6 antigen, which is associated with MN induced by non-steroidal anti-inflammatory drugs (NSAIDs) [9]. Despite the patient’s denial of chronic NSAID use, a review of their medical history reveals that he has experienced aseptic necrosis of the femoral head and chronic back pain. Moreover, the patient’s medication is administered by a family member, so it is not certain whether they used NSAIDs or not. Therefore, we cannot rule out the possibility of NSAID use with certainty.

**Table 3:** Results of laser capture microdissection and mass spectrometry

Case number	1	2	3
Target antigens	**LCM/MS count**	**LCM/MS count**	**LCM/MS count**
Protocadherin FAT 1	69	50	0
Secretory phospholipase A2 receptor	3	7	0
Proprotein convertase subtilisin/kexin type 6	0	0	26

FAT1- FAT atypical cadherin 1, LCM/MS- laser capture microdissection and mass spectrometry

We observed three cases in our study, out of which two showed high spectral counts of FAT1, measuring 50 and 69 respectively. The third case showed a moderately high spectral count for PCSK6, an antigen linked with chronic NSAID use. It’s worth noting that the two FAT1-positive patients also had baseline counts of anti-PLA2R, as observed in some other patients with HSCT-associated MN. All other known antigens, including THSD7A, EXT1/EXT2, NELL1, SEMA3B, and PCDH7, were negative.

### Results of the literature review

**Fig. 4:**
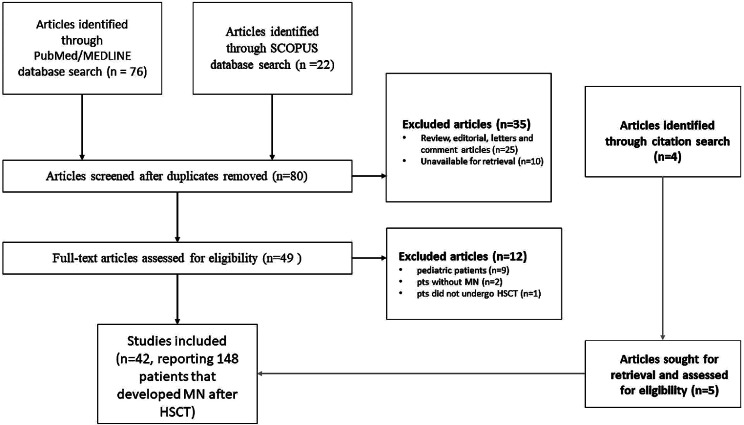
Prisma flowchart

The combined search of both PubMed/MEDLINE and SCOPUS databases yielded 98 articles. A total of forty-two articles describing 148 patients showing signs of NS after receiving HSCT and having renal biopsy findings indicative of MN have been identified, after removing duplicates and excluding articles not meeting the criteria of the literature review provided through PRISMA flow chart as shown in Fig. 4. For more details, including references to the identified articles, please refer to the Supplementary Table. The onset of NS after HSCT typically occurs after a median time of 24 months (IQR 17–36), with a median proteinuria of 7.3 g/day (IQR 4.6–13.8). Patients usually have full-blown NS. Of the published cases, 14 patients were found to be FAT1 antigen-positive, and 7 had anti-PLA2R detected through IF, serum antibody, or tissue proteomics. Our findings are in alignment with a previous review by Beyar-Katz et al. in 2016, which covered NS after HSCT. However, our review specifically focused on MN cases after HSCT, and we identified 72 additional cases. Detailed information on the clinical presentation, treatment, and outcomes of each patient is provided in the Supplementary Table.

We conducted research for specific PLA2R antigen testing in the literature. It is worth noting that out of the 24 articles included in the literature review which were published after anti-PLA2R antibody testing became available, 30% did not perform PLA2R antibody testing (see Supplementary Table).

## Discussion and conclusion

MN is one of the most common causes of adult-onset NS. It can occur as a primary disease or develop as a result of other underlying conditions. It is important to distinguish between primary and secondary forms of MN because they require different therapeutic approaches. Primary MN is the most common form and is characterized by positive anti-PLA2R antibodies in 70% of cases [7, 12, 13]. However, 30% of primary MN cases are anti-PLA2R negative, which makes it difficult to differentiate between primary and secondary forms of MN. MN is the most common cause of NS after HSCT [7, 13]. MN associated with HSCT or renal GVHD is considered secondary MN. However, considering the development mechanisms of HSCT-related MN, it is believed to be an immunologically mediated form of MN. Therefore, unlike other secondary forms of MN, immunosuppression is the preferred treatment for this specific setting [2, 5, 6, 8].

To elucidate the pathogenesis of MN after HSCT S*ethi et al.* discovered FAT1 antigen [10]. FAT1 is a large transmembrane protein belonging to the group of protocadherins, expressed in podocytes. The study showed that FAT1 antigen was present in all 14 cases of MN after HSCT that were investigated [10]. Moreover, this antigen seems to be specific to this particular subset of MN cases, since it was not found in other types of secondary MN [11].

Different types of MN can occur after HSCT. Some cases are positive for FAT1 [10], PLA2R [14–19], or NELL-1 [20], while others have a unique presentation with extensive deposits in the tubular basement membrane [19]. In our case study, we found a patient with HSCT who had a unique MN subtype, positive for PCSK6. These different subtypes of MN have varying deposit locations, IgG subtypes, and the presence and amount of C3 and C4d as shown in Table 4. In most types, deposits are global and IgG4 dominant. However, NELL1-positive cases have segmental deposit distribution and predominant IgG1. In contrast to primary MN that is usually C4d positive [21], only few cases of C4d positivity are reported in MN associated with HSCT (two PLA2R positive, one PLA2R positive with extensive TBM deposits and one with extensive TBM deposits). These positive cases concomitate thrombotic microangiopathy and endothelial injury [14, 19]. Lack of data on presence and localisation of C4d deposits in other reports prevents us from drawing further conclusions. GVHD frequently coexists with most of these subtypes. The typical clinical presentation is a nephrotic syndrome (NS) with preserved or slightly reduced kidney function.

**Table 4:** Types of membranous nephropathy after allogeneic stem cell transplantation

	MN associated with HSCT				MN with HSCT as comorbidity
	PLA2R positive	MN with extensive TBM deposits (1 case PLA2R positive)	FAT1 positive	NELL1 positive	PCSK6
Described cases	7	5	16	2	1
**Pathohistology findings**					
Deposit location	Global, subepithelial	Global, subepithelial, TBM	Global, subepithelial, and TBM in 1 Pt	Segmental, subepithelial, TBM	Global subepithelial, subendothelial, mesangial
Dominant IgG subtype	IgG4 and IgG1	IgG4	IgG4 and IgG2 in 1 Pt	IgG1	NA
C3	1+	2–3+	0–1 + Pts in literature 3 + in our Pt	1+	1+
C4d	1–3+ in 3 Pts (glomerulus and PTC) Negative in 1 Pt NA in 3 Patients	1 + in 2 Pts in PTC NA in 3 Pts	NA	NA	NA
**Clinical findings**					
Clinical presentation	NS and preserved renal function	NS and preserved renal function + acute kidney injury in 3 Pts	NS and preserved/ mildly reduced renal function	NS and preserved/mildly reduced renal function	NS and preserved renal function
Presence of GVHD	Positive in some Pts	+ in all patients(skin, liver, lungs, stomach, joints)	Negative- in our 2 Pts NA- Pts in literature	+	+ (skin, liver, eye, oral)

FAT1- FAT atypical cadhein 1, GVHD- graft versus host disease, HSCT- haematopoietic stem cell transplantation, IgG 1–4 - immunoglobulin G 1–4, PCSK6- Proprotein convertase subtilisin/kexin type 6, PLA2R- phospholipase A2 receptor, Pt- patient, PTC- peritubulary capillaries, MN- membranous nephropathy, NA- not available, NELL1- NEL-like protein 1, NS- nephrotic syndrome, TBM- tubular basement membrane

It seems that there is MN associated with HSCT and MN that occurs in patients who have a history of HSCT as comorbidity without clear association to HSCT.

Certain indications suggest HSCT-related MN or renal GVHD. In the case of our first two patients, there were a few key indicators that pointed towards HSCT-related MN. These included pathology findings that were similar to primary MN, with subepithelial electron-dense deposits and the absence of mesangial deposits. Additionally, there was a close temporal relationship between the cessation of GVHD prophylaxis [5]. The third key indicator was the detection of FAT1, a glomerular antigen that is specifically associated with HSCT.

As described by Sethi et al., specific antigen testing presents new diagnostic possibilities for the detection of post-HSCT MN linked with GVHD. This includes not only microdissection and spectral counts of FAT1 itself but also the identification of anti-FAT1 antibodies in patients’ serum. The detection of these antibodies provides further insight into the immune-mediated nature of the disease and emphasises the need for immunosuppression [10, 12, 22]. In patients who develop MN after HSCT, primary MN, secondary MN related to HSCT, or secondary MN related to other causes (such as infection, drugs, or malignant disease) should be considered as differential diagnoses.

It is crucial to differentiate between primary MN and MN related to HSCT because both of them require immunosuppression treatment while MN related to other causes do not. This distinction is particularly important in patients who have gone through HSCT since this group is often fragile and vulnerable to infectious complications. This is because they have a large burden of previously received immunosuppression, chemotherapy, or radiation therapy [20, 23].

Patients with MN after HSCT often experience serious and life-threatening infectious complications as a result of increased immunosuppression. In addition, they may also exhibit metabolic and osteo-muscular disorders such as diabetes, osteoporosis, and bone fractures which are connected to the use of corticosteroids, the mainstay of immunosuppressive treatment for MN [20]. However, exposing this subgroup of patients to unnecessary immunosuppression can suppress transplant levels to undesired level and enable haematological malignancy to flourish [24].

A personalised approach is necessary when dealing with patients who have developed MN after HSCT. This is highlighted in our third case where we observed several factors indicative of secondary MN that were unrelated to HSCT such as the absence of temporal association with cessation of immunosuppressive therapy, a long period between HSCT and onset of NS, and the presence of mesangial deposits in a kidney biopsy.

It has been observed that chronic GVHD skin changes worsen at the same time as the occurrence of NS. The relationship between a history of aGVHD or concurrent cGVHD with HSCT-related MN is not well-established. Brukamp et al. [5] state that in about half of the patients, glomerular disease is associated with concurrent GVHD, while Hu reports that concomitant cGVHD and glomerulonephritis occur in two-thirds of patients, with similar prevalence to cGVHD after HSCT without glomerulonephritis [24].

In the third case, the patient did not achieve remission with conservative treatment. The second renal biopsy showed no mesangial deposits, which led to the decision to start rituximab treatment. Several years later, specific antigen testing revealed that the patient was positive for PCSK6. This protein is usually associated with heavy and prolonged use of NSAIDs [9].

Our patient reported no use of NSAIDs despite experiencing painful femoral head necrosis. However, in the original Mayo Clinic study, 3 out of 13 patients also did not have a history of NSAID exposure [9]. It is possible that when reviewing medical history retrospectively for over-the-counter drugs like NSAIDs, they were not mentioned if not specifically asked about.

It is possible that our patient had secondary MN due to the use of NSAIDs. A small series of patients showed positive outcomes when they stopped using NSAIDs and underwent immunosuppressive therapy and conservative management [9]. We are uncertain whether the MN in our third patient would have gone into spontaneous remission over time. If it had been possible to test for specific antigens at the time, this patient probably would not have been exposed to immunosuppressive therapy emphasizing the importance of antigen testing and its impact on therapeutic decision-making.

It is interesting to note that all three patients tested negative for anti-PLA2R on serum testing. However, they were found to be vaguely positive on baseline counts on LCM/MS. This shows that different methods and samples can produce different results and interpretations and must be considered in a clinical context. Unfortunately, our study was retrospective, and we were unable to analyse the sera of our patients to detect FAT1 and PCSK6. Additionally, the lack of immunofluorescence images, confocal microscopic examination, immunohistochemistry and IgG subtyping are limitations of our study.

When discussing the outcomes related to MN associated with HSCT and GVHD, it is important to note that there are two outcome “pathways” to consider. The first pathway is related to the patient’s overall well-being and kidney function, while the second pathway is related to survivability after HSCT. If left untreated, MN can progress into end-stage renal disease (ESRD), which can lead to a loss of kidney function that may require either dialysis or a kidney transplant. However, there is currently a lack of consensus regarding the appropriate treatment approach that has a favourable risk-benefit ratio. As revealed in our review, the treatment modalities for MN are varied, ranging from corticosteroids to CYC, MMF, CsA, rituximab, and combinations of these drugs [25, 26].

This indicates that further research is necessary to create uniform guidelines for the treatment of MN associated with HSCT and GVHD. Our patients have shown a positive response to the combined treatment approach, resulting in complete remission. However, this is not always the case. Larger cohorts have reported persistence of proteinuria in 27% of patients despite immunosuppressive therapy and a high rate of mortality (6/11 patients died due to a cause unrelated to haematological disease) [10]. Additionally, up to 15% of HSCT recipients are likely to develop chronic kidney disease emphasising the importance of long-term follow-up and a multidisciplinary approach for these patients [27, 28].

After HSCT, MN is the most common cause of NS. It’s crucial to differentiate this secondary form of MN from other secondary causes to make the right therapeutic decisions. Testing for anti-FAT1 antibodies is a promising new biomarker for HSCT/FAT1-related MN, just as anti-PLA2R antibodies proved to be for PLA2R-related idiopathic MN.

### Electronic supplementary material

Below is the link to the electronic supplementary material.

Supplementary Material 1

## Data Availability

All data generated or analyzed during this report are included in this published article and PRISMA flowchart was provided in its supplementary information files. The datasets used and/or analysed during the current study available from the corresponding author on reasonable request.

## Abbreviations

ACE: Angiotensin-converting enzyme
aGVHD: Acute graft versus host disease
ALL: Acute lymphoblastic leukaemia
AML: Acute myelogenous leukaemia
CYC: Cyclophosphamide
cGVHD: Chronic graft versus host disease
CsA: Cyclosporin A
ESRD: End-stage renal disease
GBM: Glomerular basement membrane
GVHD: Graft versus host disease
HLA: Human leukocyte antigen
HSCT: Haematopoietic stem cell transplantation
IS: Immunosuppressive therapy
LMD/MS: Laser capture microdissection and tandem mass spectrometry
MMF: Mycophenolate mofetil
MN: Membranous nephropathy
MTX: Methotrexate
NS: Nephrotic syndrome
NSAID: Non-steroidal anti-inflammatory drugs
