# Supplementary material for: Specific antigen-based stratification of membranous nephropathy in patients after haematopoietic stem cell allotransplantation - a case series and literature review
Source: BMC Nephrol. 2024 Aug 8;25:254. doi: 10.1186/s12882-024-03675-y (PMC11312175; doi:10.1186/s12882-024-03675-y)
Supplement: Supplementary file 1 — Supplementary Material 1 [file 12882_2024_3675_MOESM1_ESM.docx]

Supplementary table. Overview of literature review

| AUTHORS (FIRST AUTHOR SURNAME) AND YEAR | NUMBER AND GENDER OF PATIENTS | TIME OF ONSET AFTER HSCT | NOTABLE FINDINGS | aFAT1 ab | aPLA2R ab & PLA2R | TREATMENT AND OUTCOME |
| --- | --- | --- | --- | --- | --- | --- |
| Abudayyeh, 2015 (1) | 1 (male) | 6 y | Proteinuria (5 g/d) | ND | Ab - negative | PS 60 mg/d, four weekly doses of RTX 375 mg/m^2^; reduction of proteinuria (1.1 g/d) |
| Bai, 2021 (2) | 1 (male) | 2 y | Limb soreness, proteinuria, lower extremities edema | ND | Ab - negative | mPSL 60 mg/d, 4x 0.8 g CP; achieved long-term stability |
| Beyar-Katz, 2015 (3) | 2(1 male, 1 female) | Pt1-20 mo  Pt2-16 mo | Proteinuria  Pt1- 10 g/d  Pt2-ND | ND | ND | CS and CsA |
| Byrne-Dugan, 2014 (4) | 5 (3male, 2 female) | Pt1-483 d  Pt2-268 d  Pt3-1834 d  Pt4-689 d  Pt5- 539 d | Proteinuria  Pt1-2.3 g/d | ND | Pts 1,2,3,4, - IF for PLA2R negative  Pt 5- IF for PLA2R positive | Pt1-PS 60 mg, RTX 375 mg/m^2^ weekly for four doses, CR  Pt2- died before treatment  Pt3- PS 60 mg, CR  Pt4-PS 60 mg, CR  Pt5-PS 60 mg, RTX 375 mg/m^2^ weekly for four doses, PR |
| Chan, 2008 (5) | 3 (all male) | Pt1 - 34 mo  Pt2 - 134 mo  Pt3 - 62 mo | Proteinuria:  Pt1 -24.6 g/d  Pt2 - 1.05 g/d  Pt3 - 7.6 g/d | ND | ND | Pt1 – PSL, MMF, CsA; CR  Pt2 – ACEi; normal renal function and proteinuria 0.85 g/d Pt3 – prednisolone, MMF, CsA: renal impairment |
| Cho, 2013 (6) | 12 (6 male, 6 female) | 40 mo (mean duration) | Nephrotic syndrome and proteinuria | ND | Ab - negative | CR – 6 pts  NR – 2 pts  PR – 4 pts |
| Colombo, 2006 (7) | 4 (all male) | Pt1 – 405 d Pt2 – 522 d Pt3 – 1681 d Pt4 – 466 d | Fatigue and leg edema; Proteinuria:  Pt1 – 8.9 g/d Pt2 – 12 g/d Pt3 – 5.1 g/d  Pt4 – 4 g/d | ND | ND | Pt1, 3 and 4 – PS and CsA; CR  Pt2 – CP 200 mg/d + PS 1mg/kg/d; died due to ARDS |
| Ferrannini, 2008 (8) | 1 (female) | 23 mo | Proteinuria 35.2 g/d; hypertensive crisis | ND | ND | MPSL 1g/day; relapse after 1 month; RTX 375 mg/m^2^ weekly for four doses; sustained CR |
| Fraile, 2013 (9) | 4 (2 female, 2 male) | Pt1- 17 mo  Pt2- 26 mo  Pt3- 27 mo  Pt4- 45 mo | Proteinuria with or without edema and preserved kidney function | ND | ND | Pt1-CS, CsA; CR  Pt2-CS, Tac; CR  Pt3- CS,Tac; PR  Pt4- CS, Tac; PR |
| Han, 2013 (10) | 1 (female) | 22 mo | Generalized edema, fatigue; proteinuria 5.03 g/d | ND | ND | PSL 1 mg/kg for 8 weeks; PR; relapse after 2 weeks; CsA 5 mg/kg added; CR after 4 months |
| Hiramatsu, 2016 (11) | 5 (4 male, 1 female) | Pt1 – 15 mo Pt2 – 40 mo  Pt3 – 18 mo  Pt4 – 9 mo  Pt5 – 32 mo | Proteinuria:  Pt1- 5.9 g/d  Pt2 – 9.5 g/d Pt3 – 6.5 g/d  Pt4 – 7.1 g/d  Pt5 – 13 g/d | ND | Pts 2,3 and 5 – Ab negative  Pt 1 and 4 - ND | Pt1 – ACEi; CR  Pt2 – PSL 20 mg + CsA 75 mg; CR  Pt3 – PSL 30 mg + CsA 50 mg; CR with relapse after 43 mo  Pt4 – PSL 30 mg; CR with relapse after 27 mo  Pt5 – PSL 50 mg + CsA 75 mg; CR |
| Huang, 2013 (12) | 5 (3 male, 2 female) | Pt1 – 19 mo  Pt2 – 10 mo  Pt3 – 23 mo Pt4 – 14 mo  Pt5 – 22 mo | Nephrotic syndrome; proteinuria:  Pt1 – 4.95 g/d  Pt2 – 4.56 g/d  Pt3 – 3.27 g/d  Pt4 – 6.54 g/d  Pt5 – 4.59 g/d | ND | Pts 1, 2, 3 and 5 – Ab - negative  Pt 4 - positive | Pt1 – Tac 0.1 mg/kg/d + MMF 1.5 g/d + PS 30 mg/d; PR  Pt2 – TW 60 mg/d + PS 30 mg/d; CR  Pt3 – TW 60 mg/d + MMF 1.5 g/d + PS 30 mg/d; PR  Pt4 – CsA 5 mg/kg/d + PS 30 mg/d; PR  Pt5 – Tac 0.1 mg/kg/d + PS 30 mg/d; PR |
| Ishikawa, 2011 (13) | 1 (male) | 21 mo | High fever, chest pain, abdominal pain, lower limb edema; proteinuria 7.06 g/d | ND | ND | Initial treatment: mPSL 0.5 g/d for 3d, PSL 60 mg/d; immediate improvement;  Relapse – confirmed TA-TMA; treated with PE, HD, PSL 60 mg/d, MMF up to 3000 mg/d; CR |
| John, 2023* (14) | 4 (unknown sex) | Unknown | Nephrotic syndrome | ND | 1 patient Ab - positive | Unknown |
| Kaminska, 2007 (15) | 1 (male) | 8 mo | Severe nephrotic syndrome with proteinuria up to 6.7 g/d, generalized edema, hypoalbuminemia (15g/L), hypoproteinemia (34 g/L) | ND | ND | Initially treated with CS 60 mg/d and CsA 150 mg/d; developed neuroinfection which was treated with DXM and ganciclovir/acyclovir; treatment resumed with CsA 150 mg/d and PS 50 mg/d with addition of MMF after 3 mo; pt died a month after |
| Kemper, 2007 (16) | 3 (1 male, 2 female) | Pt1 – 20 mo  Pt2 – 14 mo  Pt3 – 21 mo | Proteinuria:  Pt1 – 12.4 g/d  Pt2 – 4.8 g/d  Pt3 – 3.0 g/d | ND | ND | Pt1- initially treated with ACEi monotherapy, switched to PS 75 mg/d tapered to 50 mg/d; CR  Pt2 – pulse mPSL 500 mg/d for 3 days, followed by PS and CsA; CR  Pt3 – PS 2 mg/kg/d and CsA; CR |
| Kim, 2003 (17) | 1 (female) | 10 mo | Sudden onset of nephrotic syndrome; proteinuria 6.88 g/d | ND | ND | CsA + PS; decrease in proteinuria in 3-month time |
| Kitamura, 2019 (18) | 1 (male) | 21 mo | Bilateral leg swelling; proteinuria 10.5 g/d | ND | Ab - positive | 1 g/d mPSL, then 60 mg/d mPSL + 0.4 mg Tac |
| Kudose, 2021 (19) | 9 (6 male,3 female) | Pt1- 36 mo  Pt2- 60 mo  Pt3-unknown  Pt4- 54 mo  Pt5- 48 mo  Pt6- 48 mo  Pt7- 60 mo  Pt8- 12 mo  Pt9-unknown | Edema-1,2,3,4,5,6,8,  Proteinuria:  Pt1-6.8 g/g  Pt2- 2.2 g/g  Pt3-unknown  Pt4-3.3 g/g  Pt5-unknown  Pt6-5 g/g  Pt7-15 g/g  Pt8-5.7 g/g  Pt9-4.5 g/g | ND | Pts 1,4,5,8,9- Ab negative  Pt 6-Ab positive | Unknown |
| Lam, 2007 (20) | 1 (male) | 54 mo | Proteinuria 7.6 g/d | ND | ND | Responded to increase in immunosuppression (CsA 75 mg, PSL 40 mg, MMF 2g) |
| Masuzawa, 2017 (21) | 1 (male) | 10 mo | Progressive proteinuria due to immunosuppressant reduction; progressed to edema and pneumonia | ND | IF for PLA2R negative | 60 mg/d PS; CR |
| Miyazaki, 2003 (22) | 1 (female) | 18 mo | Nephrotic syndrome; proteinuria up to 9 g/d | ND | ND | 0.8 mg/kg mPSL; relapse 2 mo after discontinuation; mPSL reintroduced with remission after 14 days; 2^nd^ relapse treated with mPSL for 84 days; 3^rd^ relapse treated with mPSL |
| Mrabet, 2018 (23) | 1 (male) | 29 d | UTI, *S. hemolyticus* sepsis; progressive proteinuria of nephrotic range | ND | Ab- negative | RAAS; PS 1 mg/kg/d; CR |
| Nergizoglu, 1999 (24) | 1 (female) | 21 mo | Bilateral ankle edema, diarrhea; proteinuria 9.9 g/d | ND | ND | CS 8 mg/d, CsA 275 mg/d; PR |
| Numata, 2013 (25) | 1 (male) | 3 y 7 mo | Leg edema, proteinuria 5.6 g/d | ND | ND | PSL 45 mg/d; CR |
| Pilar, 2013 (26) | 4 (2 male, 2 female) | Pt1 – 17 mo  Pt2 – 26 mo  Pt3 – 27 mo  Pt4 – 45 mo | Proteinuria: Pt1 – 12.3 g/d  Pt2 – 6.77 g/d  Pt3 – 14.2 g/d  Pt4 – 27 g/d | ND | ND | Pt1- CS, CsA; CR  Pt2 – CS, Tac; CR  Pt3 - CS, Tac; PR  Pt4 - CS, Tac; PR |
| Rao, 2005 (27) | 2 (both male) | Pt1 – 12 mo  Pt2 – 14 mo | Pt1 – pedal and facial edema  Pt2 – conjunctival dryness, diarrhea, progressively increasing edema  Proteinuria:  Pt1 – 6 g/d  Pt2 – 13 g/d | ND | ND | Pt1 – PS 1 mg/kg and RTX 375 mg/m2 weekly for 4 doses; additionally added diuretics and ACEi; declined last 2 doses of rituximab; PS tapered to 10 mg/mo; PR  Pt2 – initial treatment with MMF and ACEi with no benefit; switched to RTX 375 mg/m2 weekly for 4 doses; CR |
| Ratanatharathorn, 2003 (28) | 1 (male) | 1 y | Proteinuria 20 g/d | ND | ND | Increased immunosuppression with mPSL withTac and MMF; NR after 3- and 5-months follow-up; added RTX; PR |
| Reddy, 2006 (29) | 5 (4 male, 1 female) | Pt1 – 1278 d Pt2 – 487 d Pt3 – 738 d Pt4 – 400 d  Pt5 – 385 d | Proteinuria:  Pt1 – 18 g/d  Pt2 – 5.7 g/d Pt3 – 9.1 g/d  Pt4 – 22 g/d  Pt5 – 8 g/d | ND | ND | Pt1 – mPSL + MMF; CR  Pt2 – mPSL + MMF; CR  Pt3 – CsA, mPSL, MMF, CP; CR  Pt4 – mPSL, MMF, sirolimus; PR  Pt5 – mPSL RTX; CR |
| Roy, 2023 (30) | 4 (3 male, 1 female) | Pt1 – 24 mo Pt2 – 18 mo  Pt3 – 25 mo Pt4 – 15 mo | Proteinuria:  Pt1 – 30.77 g/d  Pt2 – 3.85 g/d  Pt3 – 6.82 g/d  Pt4 – 3.14 g/d | ND | PLA2R negative; Ab negative in pts 1 and 4 | Pt1 – treatment unknown  Pt2 – repeated CS therapy due to two relapses; CR  Pt3 – treatment unknown  Pt4 – MMF + RTX; PR |
| Saddadi, 2017 (31) | 10 patients (sex unknown) | Unknown | Unknown | Unknown | Unknown | ARB, CsA, PSL; specific outcomes unknown |
| Sakai, 2023 (32) | 1 (female) | 2 y | Legs edema, proteinuria 6.65 g/d | ND | Ab – negative;  IF negative | PS 5 mg/d, RTX 375 mg/m^2^ twice; PR |
| Sethi, 2022 (33) | 14 (5 female, 9 male) | Pt1 -1.5 y  Pt2 - 4 y  Pt3 - 2 y  Pt4 - 3 y  Pt5 - NA  Pt6 - 3 y  Pt7 - 2 y  Pt8 - 2 y  Pt9 - 2 y  Pt10 - 2 y  Pt11 - 4 y  Pt12 - 2 y  Pt13 - 2 y  Pt14 - 2y | Proteinuria:  Pt1 - 3 g/d  Pt2 - 3.8 g/d  Pt3 - 3.5 g/d  Pt4 - 17 g/d  Pt5 - 22 g/d  Pt6 - 4.4 g/d  Pt7 - 7 g/d  Pt8 - 15 g/d  Pt9 - 8 g/d  Pt10 - 5.6 g/d  Pt11 - 9 g/d  Pt12 - 2 g/d  Pt13 - 4 g/d  Pt14 - 5.4 g/d | Positive in all patients | Negative in all patients | Pt3, 4, 6, 8, 12 and 13 - achieved CR although Pt12 subsequently died  Pt7, 10 and 14 achieved PR; Pt14 subsequently died  Pt1, 2, 5, 9 and 11 - died before achieving remission |
| Srinivasan, 2005 (34) | 4 (2 male, 2 female) | Pt1 – 318 d Pt2 – 326 d Pt3 – 816 d Pt4 – 1203 d | Proteinuria:  Pt1 – 30 g/d Pt2 – 22 g/d  Pt3 – 20 g/d  Pt4 – 3.0 g/d | ND | ND | Pt1 – CsA, PS; dialysis complication; died Pt2 – CsA, PS, RTX; dialysis complication; died Pt3 – CsA, PE, PS, RTX, IVIG; alive Pt4; developed TTP; died of ICH |
| Stevenson, 2005 (35) | 2 (both male | Pt1 – 22 mo  Pt2 – 25 mo | Proteinuria:  Pt1 – 14.6 g/d  Pt2 – 13.8 g/d | ND | ND | Pt1 – PS 1mg/kg/d, CsA 200 mg/d; died due to AML relapse  Pt2 – PSL 25 mg/d, perindopril and atorvastatin, CsA 200 mg/d, PR due to poor compliance |
| Sugimoto, 2007 (36) | 1 (male) | 20 mo | Proteinuria 13.1 g/d | ND | ND | PSL 40 mg/d; outcome unknown |
| Tedjaseputra, 2018 (37) | 1 (male) | 90 d | Gross peripheral edema (20 kg weight gain) and nephrotic syndrome; proteinuria over 15 g/d | ND | Ab - positive | RTX 375 mg/m^2^ weekly for four weeks; CR at 8-month follow-up |
| Terrier, 2007 (38) | 5 (3 female, 2 male) | Pt1 – 33 mo  Pt2 – 17 mo  Pt3 – 14 mo  Pt4 – 21 mo  Pt5 – 37 mo | Proteinuria:  Pt1 – 16.2 g/d  Pt2 – 8.5 g/d  Pt3 – 8.2 g/d  Pt4 – 7.9 g/d  Pt5 – 11 g/d | ND | ND | Pt1 – CS, CsA; CR with relapse after discontinuation; CR after reintroduction of CS and CsA Pt2 – CS, AZA; Died due to septic shock  Pt3 – CS, MMF, RTX, CsA; NR, deterioration of renal function Pt4 – CS, CHL; CR  Pt 5 – CS, CsA; PR with maintenance therapy |
| Troxell, 2008 (39) | 7 (4 female, 3 male) | Pt1 – 5 y Pt2 – 2 y  Pt3 – 5 y  Pt4 – 2.5 y Pt5 – 2 y Pt6 – 2.9 y  Pt7 – 1.75 y | Proteinuria:  Pt1 – 3-4g/d Pt2 – 3-4 g/d Pt3 – 15 g/d  Pt4 – 14 g/d  Pt5 – 18-21 g/d  Pt6 – 14 g/d  Pt 7 – 4 g/d | ND | ND | Pt1 – unknown  Pt2 – RTX Pt3 - unknown  Pt4 – PS, RTX  Pt5 – PS, MMF, RTX  Pt6 – PS, MMF  Pt7 - RTX |
| Wong, 2016 (40) | 4 (2 male, 2 female) | Pt1 - 18 mo  Pt2 - 1 y  Pt3 - 10 mo  Pt4 - 36 mo | Peripheral edema and proteinuria:  Pt1 - 16 g/d  Pt2 - 30.6 g/d  Pt3 - 7 g/d  Pt4 - 3 g/d | ND | Pt1- Ab negative  Pt2 – Ab negative  Pt3 and 4 - ND | Pt1 – ACEi, PSL; PR  Pt2 – PSL, ACEi, MMF; no initial response; RTX; PR Pt3 – CsA, PSL, ACEi; CR  Pt4 – PS with initial PR; Tac with CR |
| Yap, 2022 (41) | 8 (sex unknown) | Pt1 - 1.6y  Pt2 –2y  Pt3 - 4.5 y  Pt4 - 2.7 y  Pt5 – 5 y  Pt6 – 11y  Pt7 – 3y  Pt8 - 0.9y | Proteinuria:  Pt1 - 3.13 g/d  Pt2 - 5.45 g/d  Pt3 - 2.26 g/d  Pt4 - 4.42 g/d  Pt5 - 7.3 g/d  Pt6 - 1.05 g/d  Pt7 - 10.55 g/d  Pt8 - 7.62 g/d | ND | ND | Pt1 - PSL, CsA; CR  Pt2 - PSL, CsA; CR  Pt3 - PSL, CsA, MMF; CR but died later Pt4 - PSL, MMF; CR  Pt5 - MMF, CsA; CR Pt6 - RAAS blockade; CR  Pt7 - RAAS blockade; PR Pt8 - PSL, CsA, MMF; PR, died later |

Ab - negative – antibody negative, AZA – azathioprine, CHL – chlorambucil, CR – complete remission, CS- corticosteroid unspecified in original article, CsA – cyclosporine A, CP – cyclophosphamide, d - day/s, Tac – tacrolimus, HD – hemodialysis, IVIG – intravenous immunoglobulins, MMF – mycophenolate mofetil, mo - month/s, mPSL – methylprednisolone, ND – Not Determined, PE – plasma exchange, PR- partial remission, PS – prednisone, PSL – prednisolone, pt(s)– patient(s), RTX- rituximab, TA-TMA – transplantation – associated thrombotic microangiopathy, TTP – thrombotic thrombocytopenic purpura; TW – *Tripterygium wilfordii*, y - year/s, DXM – dexamethasone

y - year/s, mo - month/s, d - day/s
*patients are part of an MN NS pattern group

Supplemental References

1. Abudayyeh A, Truong LD, Beck LH, Weber DM, Rezvani K, Abdelrahim M. Membranous nephropathy in autologous hematopoietic stem cell transplant: Autologous graft-versus-host disease or autoimmunity induction? Clin Kidney J. 2015;8(4):440–4. doi:10.1093/ckj/sfv036
2. Bai MC, Wu JJ, Miao KR, Zhu JF, Mao HJ. Nephrotic syndrome in syngeneic hematopoietic stem cell transplantation recipients: A case report. World J Clin Cases. 2021;9(3):614–22. doi:10.12998/wjcc.v9.i3.614
3. Beyar-Katz O, Kruzel Davila E, Zukerman T, Fineman R, Haddad N, Okasha D et al. Nephrotic syndrome after hematopoietic stem cell transplantation: a single center experience. Minerva Med. 2015;106(6):355-7.
4. Byrne-Dugan C, Collins B, Lam AQ, Batal I, Membranous Nephropathy as a Manifestation of Graft-Versus-Host Disease: Association With HLA Antigen Typing, Phospholipase A2 Receptor, and C4d. AJKD.2014;64(6):987-993.doi:10.1053/j.ajkd.2014.09.001 Available at: https://www.sciencedirect.com/science/article/pii/S0272638614011822
5. Chan GSW, Lam MF, Au WY, Chim S, Tse KC, Lo SHK, et al. Clinicopathologic analysis of renal biopsies after haematopoietic stem cell transplantation. Nephrology. 2008;13(4):322–30. doi:10.1111/j.1440-1797.2007.00915.x
6. Cho YH, Kang SH, Kim Y, Lee MH, An GH, Chung BH, et al. De novo glomerulitis associated with graft-versus-host disease after allogeneic hematopoietic stem cell transplantation: A single-center experience. Kidney Res Clin Pract [Internet]. 2013;32(3):121–6. doi:10.1016/j.krcp.2013.07.004 Available from: http://dx.doi.org/10.1016/j.krcp.2013.07.004
7. Colombo AA, Rusconi C, Esposito C, Bernasconi P, Caldera D, Lazzarino M, et al. Nephrotic syndrome after allogeneic hematopoietic stem cell transplantation as a late complication of chronic graft-versus-host disease. Transplantation. 2006;81(8):1087–92. doi:10.1097/01.tp.0000209496.26639.cb
8. Ferrannini M, Vischini G, Di Daniele N. Rituximab in membranous nephropathy after haematopoietic stem cell transplantation. Nephrol Dial Transplant. 2008;23(8):2699–700. doi:10.1093/ndt/gfn202
9. Fraile P, Vazquez L, Caballero D, Garcia-Cosmes P, Lopez L, San Miguel J et. al. Chronic graft-versus-host disease of the kidney in patients with allogenic hematopoietic stem cell transplant. Eur J Haematol; 2013; 91:129-34.
10. Han JH, Kim HR, Kim GJ, Lim BJ, Jeong HJ, Oh HJ, et al. A case of membranous nephropathy as a manifestation of graft-versus-host disease. Kidney Res Clin Pract [Internet]. 2013;32(1):39–42. doi:10.1016/j.krcp.2012.09.008 Available from: http://dx.doi.org/10.1016/j.krcp.2012.09.008
11. Hiramatsu R, Ubara Y, Sawa N, Hasegawa E, Kawada M, Imafuku A, et al. Clinicopathological analysis of allogeneic hematopoietic stem cell transplantation-related membranous glomerulonephritis. Hum Pathol [Internet]. 2016;50:187–94. doi:10.1016/j.humpath.2015.12.005 Available from: http://dx.doi.org/10.1016/j.humpath.2015.12.005
12. Huang X, Qin W, Zhang M, Zheng C, Zeng C, Liu Z. Detection of anti-PLA2R autoantibodies and igg subclasses in post-allogeneic hematopoietic stem cell transplantation membranous nephropathy. Am J Med Sci [Internet]. 2013;346(1):32–7. doi:10.1097/MAJ.0b013e318267b5cd Available from: http://dx.doi.org/10.1097/MAJ.0b013e318267b5cd
13. Ishikawa Y, Nishio S, Sasaki H, Kudo R, Goto H, Ito M, et al. Transplantation-associated thrombotic microangiopathy after steroid pulse therapy for polyserositis related to graft-versus-host disease. Clin Exp Nephrol. 2011;15(1):179–83. doi:10.1007/s10157-010-0376-
14. John EE, Roy S, Devasia AJ, Karuppusami R, Jose N, Mani SSR, et al. Patterns of Renal Dysfunction and Profile of Kidney Biopsies in Hematopoietic Stem Cell Transplant Recipients. Glomerular Dis. 2023;3(1):9–30. doi:10.1159/000529699
15. Kaminska D, Bernat B, Vakulenko O, Kuzniar J, Tyran B, Suchnicki K, et al. Glomerular lesion and increased cytokine gene expression in renal tissue in patients with decompensated nephrotic syndrome due to chronic GvHD. Ren Fail. 2010;32(4):510–4. doi:10.3109/08860221003664256
16. Kemper MJ, Güngör T, Halter J, Schanz U, Neuhaus TJ. Favorable long-term outcome of nephrotic syndrome after allogeneic hematopoietic stem cell transplantation. Clin Nephrol. 2007;67(1):5–11. doi:10.5414/CNP67005
17. Kim KW, Yoon CH, Kay CS, Kim HJ, Suh KS, Kim SY, et al. Membranous Nephropathy after Allogeneic Hematopoietic Stem Cell Transplantation in a Patient with Aplastic Anemia : A Case Report. J Korean Med Sci. 2003;18(2):287–9. doi:10.3346/jkms.2003.18.2.287
18. Kitamura M, Hisano S, Kurobe Y, Abe S, Ota Y, Sawayama Y, et al. Membranous nephropathy with crescent after hematopoietic cell transplantation. Intern Med. 2019;58(1):91–6. doi:10.2169/internalmedicine.1251-18
19. Kudose S, Sekulic M, Mehring CJ, Santoriello D, Batal I, D'Agati V et al. NELL1-Associated Membranous Glomerulopathy After Hematopoietic Stem Cell Transplantation. Kidney Int Rep. 2021;6(7):1992-5. doi:10.1016/j.ekir.2021.04.033 Available from: https://www.kireports.org/article/S2468-0249(21)01146-3/fulltext#appsec1
20. Lam MF, Au WY, Tse KC, Chan TM, Chan GSW, Chan KW, et al. Late Onset Membranous Nephropathy Complicating Donor Lymphocyte Infusion for Leukaemia Relapse After Allogeneic Stem Cell Transplantation. Am J Hematol. 2007;82:327–33. doi:10.1017/9781108772297.029
21. Masuzawa N, Nishimura A, Kitani T, Tamagaki K, Sugitani M, Nagoshi H, et al. A Case of the nephrotic syndrome in bone marrow transplantation recipient, histologically showing overlapped glomerular lesions of thrombotic microangiopathy and membranous nephropathy. Pathol Int. 2017;67(12):620–5. doi:10.1111/pin.1258
22. Miyazaki Y, Mori Y, Kishimoto N, Matsumoto N, Zen K, Amakawa R, et al. Membranous Nephropathy Associated with Donor Lymphocyte Infusion following Allogeneic Bone Marrow Transplantation. Int J Hematol. 2003;78(3):262–5. doi:10.1007/BF02983805
23. Mrabet S, Aicha N Ben, Abdessayed N, Mokni M, Achour A. Membranous nephropathy succeeding autologous hematopoietic stem cell transplant: A case report. BMC Nephrol. 2018;19(1):1–4. doi:10.1186/s12882-018-0855-z
24. Nergizoglu G, Keven K, Ates K, Ustun C, Tulunay O, Beksac M, et al. Chronic graft-versus-host disease complicated by membranous glomerulonephritis. Nephrol Dial Transplant. 1999;14:2461–3.
25. Numata A, Morishita Y, Mori M, Saito O, Takemoto F, Ando Y, et al. De novo postallogeneic hematopoietic stem cell transplant membranous nephropathy. Exp Clin Transplant. 2013;11(1):75–8. doi:10.6002/ect.2012.0078
26. Pilar F, Lourdes V, Dolores C, Pedro GC, Lucia L, Jesus SM, et al. Chronic graft-versus-host disease of the kidney in patients with allogenic hematopoietic stem cell transplant. Eur J Haematol. 2013;91(2):129–34. doi:10.1111/ejh.12149
27. Rao PS. Nephrotic syndrome in patients with peripheral blood stem cell transplant. Am J Kidney Dis. 2005;45(4):780–5. doi:10.1053/j.ajkd.2005.01.003
28. Ratanatharathorn V, Ayash L, Reynolds C, Silver S, Reddy P, Becker M, et al. Treatment of chronic graft-versus-host disease with anti-CD20 chimeric monoclonal antibody. Biol Blood Marrow Transplant. 2003;9(8):505–11. doi:10.1016/S1083-8791(03)00216-7
29. Reddy P, Johnson K, Uberti JP, Reynolds C, Silver S, Ayash L, et al. Nephrotic syndrome associated with chronic graft-versus-host disease after allogeneic hematopoietic stem cell transplantation. Bone Marrow Transplant. 2006;38(5):351–7. doi:10.1038/sj.bmt.1705446
30. Roy G, Iordachescu I, Royal V, Lamarche C, Ahmad I, Nadeau-Fredette AC, et al. Kidney Biopsy Findings Among Allogenic Hematopoietic Stem Cell Transplant Recipients With Kidney Injury: A Case Series. Kidney Med [Internet]. 2023;5(7):100674. doi:10.1016/j.xkme.2023.100674 Available from: https://doi.org/10.1016/j.xkme.2023.100674
31. Saddadi F, Alidadi A, Hakemi M, Bahar B. Nephrotic syndrome after hematopoietic stem cell transplant: Outcomes in Iran. Exp Clin Transplant. 2017;15:90–2. doi:10.6002/ect.mesot2016.O70
32. Sakai T, Uchida T, Iwama S, Sugisaki K, Yamada M, Inamoto Y, et al. Chronic Graft-versus-host Disease-associated Membranous Nephropathy Following Bone Marrow Transplantation, Successfully Treated with Rituximab. Intern Med. 2023;62(2):269–73. doi:10.2169/internalmedicine.9655-22
33. Sethi S, Madden B, Moura MC, Nasr SH, Klomjit N, Gross LA, et al. Hematopoietic Stem Cell Transplant-Membranous Nephropathy Is Associated with Protocadherin FAT1. J Am Soc Nephrol. 2022;33(5):1033–44. doi:10.1681/ASN.2021111488
34. Srinivasan R, Balow JE, Sabnis S, Lundqvist A, Igarashi T, Takahashi Y, et al. Nephrotic syndrome: An under-recognised immune-mediated complication of non-myeloablative allogeneic haematopoietic cell transplantation. Br J Haematol. 2005;131(1):74–9. doi:10.1111/j.1365-2141.2005.05728.x
35. Stevenson WS, Nankivell BJ, Hertzberg MS. Nephrotic syndrome after stem cell transplantation. Clin Transpl. 2005; 19(1):141-4. [doi:10.1111/j.1399-0012.2004.00294.x](https://doi.org/10.1111/j.1399-0012.2004.00294.x)
36. Sugimoto T, Tanaka Y, Sakaguchi M, Osawa N, Tanaka Y, Uzu T, et al. A case of post-allogeneic haematopoietic stem cell transplantation membranous nephropathy. Nephrol Dial Transplant. 2007;22(11):3360–2. doi:10.1093/ndt/gfm451
37. Tedjaseputra A, McLornan D, McCormick J, Raj K, de Lavallade H, Potter V, et al. Anti-type M phospholipase A2 receptor antibody-positive membranous nephropathy as a part of multi-system autoimmune syndrome post-allogeneic stem cell transplantation. Intern Med J. 2018;48(4):481–3. doi:10.1111/imj.13762
38. Terrier B, Delmas Y, Hummel A, Presne C, Glowacki F, Knebelmann B, et al. Post-allogeneic haematopoietic stem cell transplantation membranous nephropathy: Clinical presentation, outcome and pathogenic aspects. Nephrol Dial Transplant. 2007;22(5):1369–76. doi:10.1093/ndt/gfl795
39. Troxell ML, Pilapil M, Miklos DB, Higgins JP, Kambham N. Renal pathology in hematopoietic cell transplantation recipients. Mod Pathol. 2008;21(4):396–406. doi:10.1038/modpathol.3801011
40. Wong E, Lasica M, He SZ, Bajel A, Roberts AW, Mason KD, et al. Nephrotic syndrome as a complication of chronic graft-versus-host disease after allogeneic haemopoietic stem cell transplantation. Intern Med J. 2016;46(6):737–41. doi:10.1111/imj.13098
41. Yap DYH, Lie D, Lau T, Tang A, Chan G, Chan TSY, et al. Clinico-pathological correlations and outcomes of de novo glomerular diseases in patients after haematopoietic stem cell transplantation . Clin Kidney J. 2022;16(6):976–84. doi:10.1093/ckj/sfac264
